# Offender assessment, case planning, and referral to community-based treatment: effects of a structured process improvement initiative

**DOI:** 10.1186/1940-0640-10-S1-A70

**Published:** 2015-02-20

**Authors:** Wayne N Welsh, Hsiu-Ju Lin, Roger H Peters, Lynda AR Stein, Sami Abdel-Salam

**Affiliations:** 1 Department of Criminal Justice, Temple University, Philadelphia, PA, 19122, USA; 2 School of Social Work, University of Connecticut, Hartford, CT, 06134, USA; 3 Louis de la Parte Florida Mental Health Institute, University of South Florida, Tampa, FL, 33612, USA; 4 Cancer Prevention Research Center, University of Rhode Island, Kingston, RI, 02881, USA; 5 Criminal Justice Department, West Chester University, West Chester, PA, 19383, USA

## Background

CJDATS-2 (Criminal Justice Drug Abuse Treatment Systems), a 5-year multisite national research collaborative funded by the National Institute on Drug Abuse (NIDA), targeted implementation of evidence-based approaches for assessing and treating drug abuse within criminal justice settings. The Organizational Process Improvement Intervention, one of the major projects within the collaborative, was an intervention designed to improve the assessment of prisoners, the development of case plans for community services, the transfer of this information to community treatment agencies, and the use of the case plans by community treatment agencies that provide the recommended services. A local change team (LCT) consisting of criminal justice and community treatment staff and a facilitator conducted an organizational needs assessment and then identified and implemented targeted process improvement goals over an 18 to 24-month period.

## Methods

A Staff Perceptions of Assessment survey was administered to correctional and treatment personnel (n = 1509) at 21 sites randomly assigned to an early (experimental) or delayed (control) start condition. We hypothesized that the experimental sites would show greater improvement in staff perceptions of assessment practices than control sites (H1), and that any observed experimental effects would be sustained during a 3-month follow-up period (H2). Hierarchical linear models with repeated measures were used to examine impacts on four dimensions of assessment: measurement and instrumentation (MI), integration with the case plan (ICP), conveyance and utility (CU), and service activation and delivery (SAD). Organizational characteristics were examined as covariates to control for differences across the 21 research sites. Two planned contrasts were used to test the intervention and sustainability hypotheses. As opposed to omnibus F-tests for interaction terms, planned contrasts isolate the appropriate study condition x interval mean comparisons and provide more statistically powerful tests. Sequential Bonferroni comparisons adjusted for inflated type I error rates due to multiple comparisons. Semistructured interviews were conducted with members of the LCT and agency staff (n = 213), who were potentially affected by changes to the assessment process.

## Results

Significant intervention and sustainability effects were found for MI, ICP, and SAD. Contrary to expectations, no significant effects were found for CU. Qualitative analyses suggested that the ability of the LCT to impact intra-agency policy may in part explain the stronger implementation outcomes in the MI and ICP domains. The CU and SAD domains were more dependent on inter-agency activities, making it more difficult to enact proposed changes.

## Conclusions

It is feasible to use implementation strategies to improve evidence-based assessment practices for offenders exiting correctional systems and re-entering the community. Intra-agency assessment activities that were more directly under the control of correctional agencies were implemented more effectively. Activities in domains that required cross-system collaboration were not as readily implemented, although longer follow-up periods might afford detection of stronger effects. Such collaboration may require greater trust and reciprocity between agencies, which takes time to develop. Further efforts to improve interagency collaboration may be a prerequisite for improved conveyance and use of assessment information.

